# Serous Borderline Ovarian Epithelial Type Tumors of Testis in A Young Man with Only Mass Resection: A Case Report

**DOI:** 10.30699/IJP.2023.547886.2880

**Published:** 2023-10-15

**Authors:** Mandana Rahimi, Yasaman Moradi, Farhood Khaleghimehr

**Affiliations:** 1 *Department of Pathology, Hasheminejad Kidney Center, School of Medicine, Iran University of Medical Sciences, Tehran, Iran*; 2 *Department of Urology, Hasheminejad Kidney Center, School of Medicine, Iran University of Medical Sciences, Tehran, Iran*

**Keywords:** Papillary carcinoma, Serous, Testis

## Abstract

Tumors of the ovarian epithelial type of testis are an infrequent entity. We report a case of borderline serous tumor in an 18-year-old male who presented with a right testicular mass, clinically suspicious of carcinoma. After right inguinal exploration, two pedunculated para-testicular masses were identified in the appendix of the right testis and epididymis. The histological features were as complex papillary structures lined by columnar cells with mild to moderate pleomorphism. Microscopically, features of borderline serous testicular tumors are identical to the morphology of the same tumors encountered in the ovarian counterparts. These tumors usually reveal papillae with fibrovascular cores lined by stratified cuboidal to columnar epithelium. This case highlights a need for clinicians and pathologists to be aware of this infrequent entity and improve the best patient management attitude. Serous epithelial tumors are common ovary tumors but are very rare entities in the testis. These tumors originate from the remnant of Mullerian ducts or Mullerian metaplasia of tunica vaginalis and are nonaggressive, even associated with extra ovarian spread, and have outstanding prognosis. A review of the literature has shown nearly fifty reported cases worldwide, and most of the cases occur in young to middle-aged adults.

## Introduction

Tumors of the ovarian epithelial type of testis are an infrequent entity. According to the reported cases, the age range is 14 to 68 years, usually observed as a scrotal enlargement (1). The most commonly reported are serous tumors, with the majority of the cases to be the borderline type (2,3).

## Case Presentation

Here, we report an 18-year-old man complaining of fullness and painless mass in the right hemiscrotum. He was a hairdresser with no history of any medical diseases. On physical examination, the patient had a right hemiscrotal mass from 4 years ago, without pain or increase in size. The blood biochemistry was within normal limits: BUN: 13 mg/dL, Creatinine 1 mg/dL, Sodium: 135 mEq/L, Potassium: 4.0 mEq/L. Serum tumor markers were: human chorionic gonadotropin (hCG):<2 U/L (normal 0-2), alpha-fetoprotein (AFP): 1.2 ng/mL (normal <8.1), lactate dehydrogenase (LDH): 226 U/L (normal<480).

**Fig. 1:**
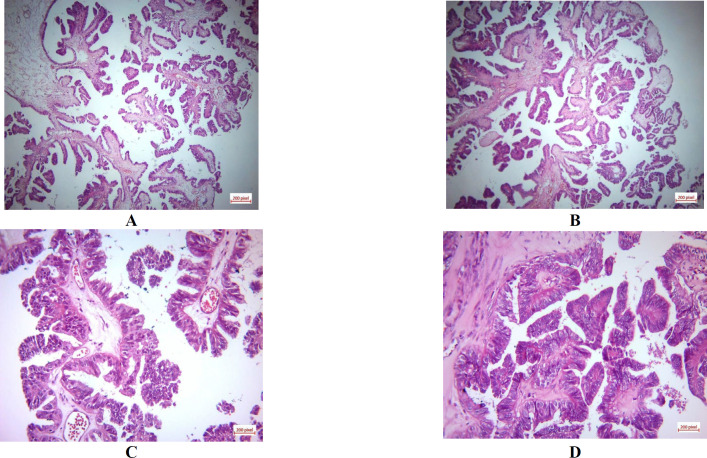
**Histology H&E:** (A,B) serous papillary tumor: complex papillary structures lined by columnar cells (high magnification), C) showing papillary arrangement and a hobnail-like pattern of the tumoral cells with mild to moderate pleomorphism, vesicular nuclei; and eosinophilic cytoplasm (x20), D) showing micropapillary pattern (x40)

Through ultrasonography, the s mass proved to be cystic and solid. A homogenous lobulated mass measuring 23 × 21 × 20 mm with an exophytic growth pattern originating from the right epididymis and some foci of peripheral calcification around was seen (Figure 1).

Under the hypothesis of a malignant testicular tumor, an inguinal incision was performed. The macroscopic inspection of the surgical specimen consisted of a right testicular appendix mass, presented a size of 2 × 2 × 1.8 cm, a right epididymal mass size of 0.3 × 0.3 × 0.2 cm, and a hydrocele contained approximately 50 mL yellowish fluid. The background testicular tissue seemed macroscopically normal.

Due to pedunculated nature of the tumors and small size of the tumors, only the entire mass was resected, processed and submitted for histological examination. Histologically, complex papillary structures lined by columnar cell with mild to moderate pleomorphism, vesicular nuclei, a few nucleoli, and eosinophilic cytoplasm were identified. No mitotic figures, necrosis, or stromal invasion were noted.

**Fig. 2:**
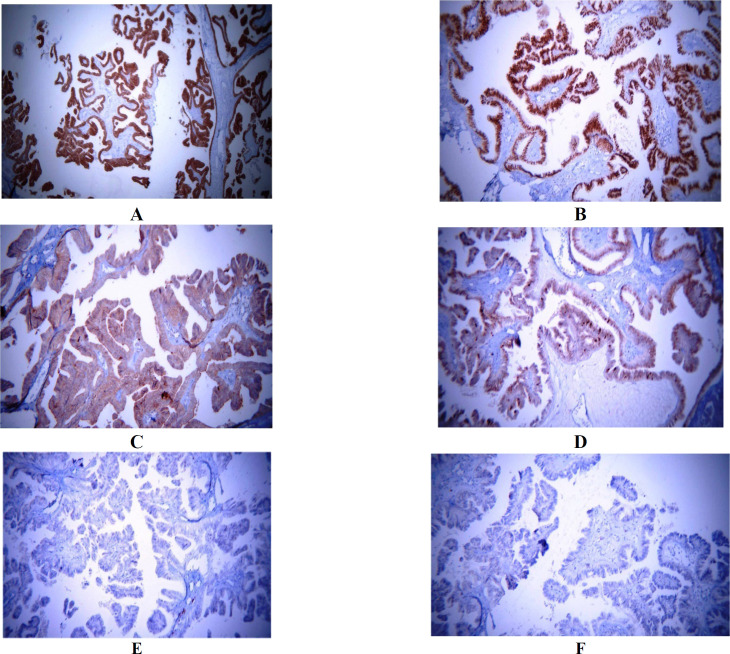
**Immunohistochemi** **cal** **staining of** **the s** **erous borderline tumor of the testis**: The lining epithelial tumoral cells express CK7 (a; ×200); Estrogen receptor was positive in more than 90% of the tumoral cells (b; x200), CA125 positive staining in the tumoral cells (c; x200). The Ki67 staining exhibits low proliferative activity (d; x200), Calretinin negative staining (E; x200).CEA negative staining (F; x400)

Through immunohistochemical staining, CA125, CK7, ER, and CK5/6 showed to be positive in the tumoral cells. ER was positive in more than 90% of the tumoral cells, and Ki-67 was positive in about 5% of the tumoral cells. Calretinin and CEA were negative in the tumoral cells (Figure 2).

After the removal of the tumor, the patient was followed up, and no recurrence or metastasis has occurred to date. We report here of an 18-year-old male resented with a suspicious malignant tumor in the right paratesti.

## Discussion

According to the 2016 WHO classification of testis tumors, ovarian epithelial-type tumors are categorized in the set of miscellaneous tumors of the testis. These tumors are more normally para-testicular rather than testicular. All the acknowledged types of epithelial tumors morphologically identical to the ovarian surface epithelial tumors have been reported in testicular and paratesticular tissues. The most frequently reported are serous tumors, with most cases being of the borderline type (3,4).

Other ovarian tumors have also been described in testicular and paratesticular tissues, including mucinous, endometrioid, clear cell, transitional (Brenner) tumors, and serous carcinoma (5). Borderline serous tumors are likely not to recur or metastasize. Contrariwise, serous carcinoma can metastasize and is related to an unfavorable prognosis (6). 

Consequently, extensive sampling of all cases of borderline tumors is imperative. The borderline serous tumor we report here was processed in its totality for histological examination.

No stromal invasion was identified, and there was no evidence of recurrence or metastasis. The histogenesis of these epithelial tumors in testicular and paratesticular tissues is a substance of significant discussion. Several hypotheses exist in the literature. Some propose that these tumors arise from the remnants of Mullerian ducts found in the male appendix testis, epididymis, and connective tissue between the testis, epididymis, and spermatic cord. Others suggest that the bases of origin are the Mullerian metaplasia of the surface lining mesothelium and metaplasia of mesothelium surrounded by testicular parenchyma. The latter clarifies the pathogenesis of such tumors rising in the tunica vaginalis (4,5,6).

These tumors typically come about in young and middle-aged adults. The mean age of patients with borderline tumors is 56 years (range 14-77 years), and for invasive tumors, 31 years (range 16-42 years) (7-9). Dull pain, swelling, palpable mass, and accompanying hydrocele are communally presenting signs and symptoms. Often, cancer antigen 125 (CA-125) levels may be raised. On gross examination, borderline tumors of the testicular and paratesticular tissues are almost always cystic with a thin fibrous capsule, while invasive carcinomas are typically non-cystic and further infiltrative (9).

Hardly any authors are confident that serous borderline tumors of ovarian counterparts with micropapillary growth patterns tend to have advanced stages, microinvasion or lymph node involvement, and worse prognosis. However, most believe that micropapillary growth pattern is not a worse prognostic factor.

Borderline serous tumors of the testis are inclined not to recur or metastasize, but the prognosis of ovarian-type epithelial tumors is not precise because of their rarity (10).

The scarcity of these tumors in the testis may lead to difficulty and confusion regarding their diagnosis. Such tumors are in the differential diagnosis of other tumor types, such as mesothelioma. Some histological criteria are supportive in discriminating borderline serous tumors from mesothelioma. Localized and non-destructive gross appearance, ciliated lining epithelium and psammoma bodies, and positivity for cytokeratin CK7, estrogen, and negativity for CK20 and calretinin all favor the diagnosis of serous tumors. Positivity to calretinin favors the diagnosis of mesothelioma (6,8).

In summary, borderline serous tumors of the testis and paratestis are extremely rare; therefore, clinicians and pathologists should keep this entity in mind when dealing with unusual testicular tumors. The identification of invasive components in an otherwise borderline tumor is of prognostic significance; as a result, detailed sampling should be assumed. The prognosis for patients after complete excision of borderline tumors is outstanding, according to a few previous studies and case reports. Still, in our study, the urologist surgeon planned just to resection the mass, not orchiectomy such as in other reported cases yet, because of the pedunculated nature of the tumor and the small size of the tumor. It was the difference between this case and another case report of this type of tumor. So, no tumor recurrence was found on the next year's follow-up sonography imaging and assessment of tumor markers. Nevertheless, clinical follow-up is mandatory for at least several years.

Additionally, insufficient clinical information and experience in managing such patients due to the tumors' rarity highlight the need to develop the best management approach for patients with borderline serous tumors of the testis and paratestis.

## Conclusion

None.

## Funding

 None.

## Conflict of Interest

There is no conflict of interest.
